# Early reintervention for hemostasis following open abdominal aortic aneurysm repair using Ifabond surgical glue

**DOI:** 10.1016/j.jvscit.2025.101999

**Published:** 2025-10-10

**Authors:** Christoph Bacri, Alexis Zhan, Kheira Hireche, Pierre Alric, Ludovic Canaud

**Affiliations:** a Department of Thoracic and Vascular Surgery, Arnaud de Villeneuve Hospital, Montpellier, France; b Physiology and Experimental Medicine of the Heart and Muscles, University of Montpellier, CNRS, INSERM, CHU Montpellier, Montpellier, France

**Keywords:** Open aortic surgery, AAA, Abdominal aneurysm, Reintervention, Hemostasis, Surgical glue, Ifabond

## Abstract

**Objective:**

The aim of this study is to evaluate the rate and type of early reintervention and outcomes after open aortic surgery in a high-volume aortic center and to determine the impact of Ifabond surgical glue as an hemostatic barrier.

**Methods:**

All patients who underwent aortic surgery at a single center between 2021 and 2023 were reviewed. The primary end point was a comparison of early reintervention rates for hemostasis between patients with and without Ifabond surgical glue application. Secondary end points included comparisons of early reintervention, early outcomes, and hemoglobin management. Additional subgroup analyses using the same variables were performed to assess the impact of the type of repair.

**Results:**

From January 2021 to December 2023, 383 patients underwent open aortic surgery at a single tertiary referral center. There were 159 patients who met the inclusion criteria, with surgical glue used in 130 cases (82%). Among these patients, 82 (63%) underwent aortoaortic repair. Five patients (3%) died, and six (4%) required early reintervention: three for hemostasis, two for thrombectomy, and one for both. No cases of bowel ischemia were reported. Although not statistically significant, there was a trend toward lower reintervention rates for bleeding in the glue group (1.5% vs 6.9%; *P* = .15), but there was no impact on transfusion requirements (18% vs 14%; *P* = .79). No difference was observed in overall reintervention rates (3.1% vs 6.9%; *P* = .31) or early outcomes. Comparing repair types (aortoaortic vs aortoiliac) revealed significant differences in operative duration (123.0 ±38.7 vs 149.0 ± 38.6 minutes; *P* < .001), with aortoaortic repairs being shorter and more commonly performed via the retroperitoneal approach (65% vs 10%; *P* < .001).

**Conclusions:**

Application of glue appears to decrease reintervention rates for hemostasis and postoperative hemoglobin loss. Further prospective studies are warranted to better define the role of Ifabond surgical glue in abdominal aortic repair.

**Article Highlights**

• **Type of Research:** Retrospective study
• **Key Findings:** From 2021 to 2023, 383 patients underwent aortic surgery at a single center. Among these, 159 patients underwent abdominal aortic aneurysm repair, with 130 benefiting from the application of Ifabond surgical glue on the anastomosis. During the early follow-up period (30 days), five patients (3%) died, and six (4%) required reintervention: three for hemostasis, two for thrombectomy, and one for both. A comparison of early outcomes between those with and without glue applied to the anastomosis shows a trend favoring the use of glue, with fewer reinterventions for hemostasis and decreased blood loss, although there was no impact on transfusion requirements.
• **Take Home Message:** The use of Ifabond surgical glue on aortic anastomoses in abdominal aortic repair appears to decrease the rates of reintervention and transfusion.

Open aortic surgery has traditionally been the treatment of choice for various aortic pathologies. Although endovascular repair has become prominent in managing aortic disease, especially for aneurysm repair,^1^ open repair is still frequently required in cases of unsuitable aortic anatomy for endovascular aortic repair (EVAR), in instances of endovascular failure and in cases of aortic and/or prosthetic infections.

Early reinterventions are primarily necessitated by digestive complications (36%) and bleeding (21%).^2^ It is crucial to identify and address early complications promptly, with medical management or early reintervention when needed. However, early reintervention is associated with greater mortality, underscoring the need to limit these events through optimized perioperative care and meticulous intraoperative hemostasis.

To prevent reinterventions for bleeding in aortic surgery, various hemostatic aids, such as Surgicel and surgical adhesives, are available. Although these products are commonly used in cardiac surgery, their application is increasing in abdominal aortic surgery and may reduce perioperative complications. Nevertheless, particularly in the context of abdominal aortic aneurysm repair, there is limited literature on the use of surgical glue.

The objective of this study was to describe the rate and types of early reintervention after open abdominal aortic aneurysm repair, comparing outcomes with and without the use of surgical glue in a high-volume center.

## Methods

### Ethics

This study was approved by the local institutional review board. No patients declined to participate.

### Patients

From January 2021 to December 2023, all patients undergoing open abdominal aortic surgery at a single tertiary referral center were retrospectively reviewed. Inclusion criteria comprised all patients treated for abdominal aortic or aortoiliac aneurysms by open aortoaortic or aortoiliac repair. Exclusion criteria included thoracoabdominal aneurysm, paravisceral aneurysm, pararenal aneurysm, abdominal aneurysm repair with additional visceral bypass, endoaneurysmorrhaphy, aortic occlusive disease, aortovisceral bypass, aortic infections, hybrid procedures, associated femoropopliteal revascularization, and emergency procedures.

Data were retrospectively collected on patient demographics (age, sex, comorbidities), operative details (type of surgery, duration), and postoperative outcomes (transfusion requirements, hemoglobin levels, complications, reinterventions, and mortality). Early outcomes were defined as those occurring within the first 30 days postoperatively.

### End points

The primary end point was the comparison of early surgical reintervention rates for hemostasis between patients with glued anastomoses and those without. Secondary end points included the 30-day reintervention rate for any cause, hemoglobin levels and management, complications, reinterventions, and mortality, comparing patients who received glue vs those who did not. Subanalyses were conducted among patients who received glue, assessing the same variables to examine differences between types of aortic reconstruction: aortoaortic vs aortoiliac.

### Surgical technique

During the initial procedure, patients underwent either a median laparotomy or a left retroperitoneal approach. Intravenous heparin (50 IU/kg) was administered after complete dissection and before clamping. No additional antiplatelet therapy was given intraoperatively, and anticoagulation was not reversed at the end of the procedure. After aortic cross-clamping, the aneurysm sac was opened and the lumbar arteries were ligated with Prolene 4/0. The pararenal aorta was completely dissected, and the proximal neck was prepared. A Teflon felt was not used systematically, only in cases of a thin and friable aorta. All prosthetic grafts were silver-coated woven Dacron grafts, either straight or bifurcated.

Aortic sutures (single running sutures) were performed with Prolene 3/0 or 2/0, and sutures on the iliac arteries with Prolene 3/0 or 4/0. Pledgets were used when additional hemostatic support was required. The inferior mesenteric artery was systematically reimplanted into the prosthetic graft when patent. The surgical glue used in this study is always Ifabond (Peters Surgical), a synthetic surgical adhesive composed of 98% n-hexyl cyanoacrylate. Complete resorption occurs within 6 to 12 months, according to the information provided by the manufacturer. The glue is applied over the sutures through a catheter, and 1 minute is allowed to ensure proper polymerization. Surgicel is applied in cases of minor oozing. No other hemostatic agent is used.

Before reintervention, in cases of suspected infection or bowel ischemia, a computed tomography (CT) scan is performed. The reintervention is carried out through a median laparotomy. In cases of ischemia, an echo-Doppler or CT scan is used to guide the procedure, which may involve thrombectomy and/or bypass. Reinterventions are most often performed through the Scarpa's triangle and the femoral artery. In cases of bleeding, it is usually externalized through the Redon drains. A CT scan is not routinely performed before reintervention, which is generally undertaken through the initial surgical approach (retroperitoneal or median laparotomy). The hematoma is evacuated, and the anastomoses as well as the residual aneurysm sac are inspected for potential late bleeding from lumbar arteries. Additional Prolene stitches are placed if needed. The cavity is irrigated with warm saline solution. The abdominal wall is carefully inspected, particularly in retroperitoneal approaches where the parietal peritoneum has been widely mobilized. Any bleeding is cauterized, and veins are clipped as necessary.

### Clarification of terms used

Surgical reintervention is classified based on intraoperative findings; if no apparent issue is identified, it is classified as an abdominal exploration. The date of reintervention is recorded as the date of the first reintervention if multiple surgeries are needed. No distinction is made between patients on single and dual antiplatelet therapy, as therapy is reduced to single antiplatelet therapy (aspirin) before elective surgery. Oral therapeutic anticoagulation is discontinued preoperatively and may be bridged with injections (low-molecular-weight heparin or unfractionated heparin depending on renal function), as per anesthesiologist recommendations. Aortoiliac surgery includes both aortobi-iliac bypass and aortoiliac with complete iliac bifurcation replacement. Hemoglobin variation is analyzed as a ratio to the preoperative level, calculated as follows: (Hb_x – preoperative Hb)/preoperative Hb. Acute kidney injury was defined as a transient increase in serum creatinine of at least 26.5 μmol/L above the preoperative level (KDIGO stage 1).

### Follow-up

Patients underwent routine laboratory testing daily for the first 3 days, then every 2 days, until hospital discharge. Clinical evaluations were conducted daily throughout the hospital stay. All surviving patients underwent postoperative surveillance imaging, including an ultrasound before hospital discharge. Routine CT scans were not performed before discharge or during follow-up. Long-term follow-up is not reported here, because our focus was on early reintervention for hemostasis after an open abdominal aortic aneurysm repair.

### Statistical analysis

Continuous variables are presented as median and interquartile range or mean ± standard deviation and were compared using the Mann-Whitney *U* test or Welch's *t* test, respectively. Categorical data are presented as percentages and were compared using the χ^2^ or Fisher's exact test (two tailed). All *P* values of 0.05 or less were considered statistically significant. All statistical analyses were performed using STATA version 14.2 (StataCorp LP).

## Results

### Overall results and comparison between the use of glue or not

#### Patient distribution

**Fig 1:**
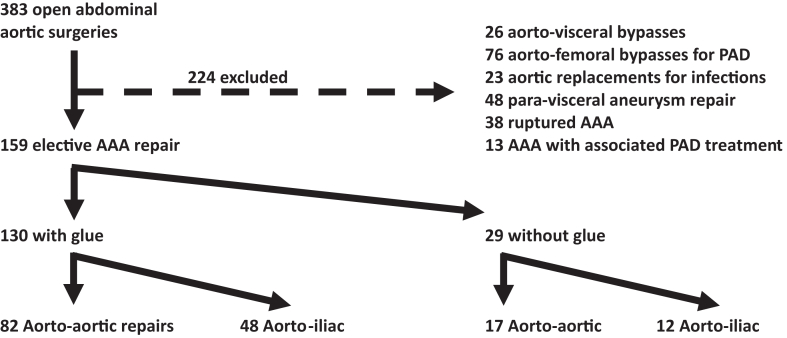
Flowchart. *AAA*, abdominal aortic aneurysm; *PAD*, peripheral arterial disease.

From January 2021 to December 2023, 383 patients underwent open abdominal aortic surgery at a tertiary referral center (aortic center). Of these, 224 patients met the exclusion criteria. Data from 159 patients were retrospectively collected and analyzed. Fig 1 provides a flowchart of patients who underwent open aortic surgery.

#### Demographics

In 130 patients (82%), Ifabond surgical adhesive was used for the anastomosis. The demographics of the patients were similar between those receiving glue and those who did not, except for age: patients receiving glue were younger (70 years vs 74 years; *P* = .04). Preoperative details are presented in Table I. After the drug switch, no hypocoagulable or hypercoagulable states were observed.

**Table I:** Demographic details of all included patients and comparison if use of glue

Variable	Overall (n = 159)	With glue (n = 130)	Without glue (n = 29)	*P* value
Age, years	71 ± 7.2	70 ± 6.5	74 ± 9	**.04**
Male sex	149 (92)	121 (93)	28 (97)	.69
Hypertension	91 (57)	77 (59)	14 (48)	.28
Atrial fibrillation	12 (8)	9 (7)	3 (10)	.46
Coronary disease	52 (33)	45 (35)	7 (24)	.28
Renal insuffiency^a^	27 (17)	23 (18)	4 (14)	.79
Diabetes mellitus	36 (23)	28 (22)	8 (28)	.48
PAD	18 (11)	115 (88)	26 (90)	1
Abdominal surgery	66 (42)	53 (41)	13 (45)	.69
ASA 3 or 4	85 (53)	69 (53)	16 (55)	.84
APT	124 (78)	103 (79)	21 (72)	.42
Oral anticoagulation	18 (11)	15 (12)	3 (10)	1
DOAC	17 (11)	14 (11)	3 (10)	1
VKA	1 (1)	1 (0.8)	0 (0)	1

*APT,* Antiplatelet therapy; *ASA,* American Society of Anesthesiologists; *DOAC,* direct oral anticoagulants; *PAD,* peripherical arterial disease; *VKA*, vitamin K antagonists.

Boldface entries indicate statistical significance.

Values are mean ± or number (%).

a Glomerular filtration rate of <30 mL/min/1.73 m^2^.

#### Operative details

Operative details are shown in Table II. Ifabond was used significantly more frequently in cases of retroperitoneal approach compared to median laparotomy (45% vs 21%; *P* = .02). The duration of surgery (from incision to skin closure) was significantly shorter when glue was used: 127 minutes vs 161 minutes (*P* < .001).

**Table II:** Operative details of all included patients and comparison if use of glue

Variable	Overall (n = 159)	With glue (n = 130)	Without glue (n = 29)	*P* value
Maximal diameter, mm	55 [7.5]	55 [6.8]	55 [11]	.3
Including iliac artery	33 (21)	26 (20)	7 (24)	.62
Aortic-aortic repair	99 (62)	82 (63)	17 (59)	.65
Aortic-iliac repair	60 (38)	48 (37)	12 (41)	.65
Laparotomy	94 (59)	72 (55)	22 (76)	**.04**
Retroperitoneal approach	65 (41)	58 (45)	6 (21)	**.02**
Duration of surgery, minutes	137 [59]	127 [56]	161 [46]	**<.001**
Duration of clamping, minutes	52 [24]	52 [23]	62 [27]	**<.01**

Boldface entries indicate statistical significance.

Values are mean ± or number (%) or median [interquartile range].

#### Early follow-up

**Fig 2:**
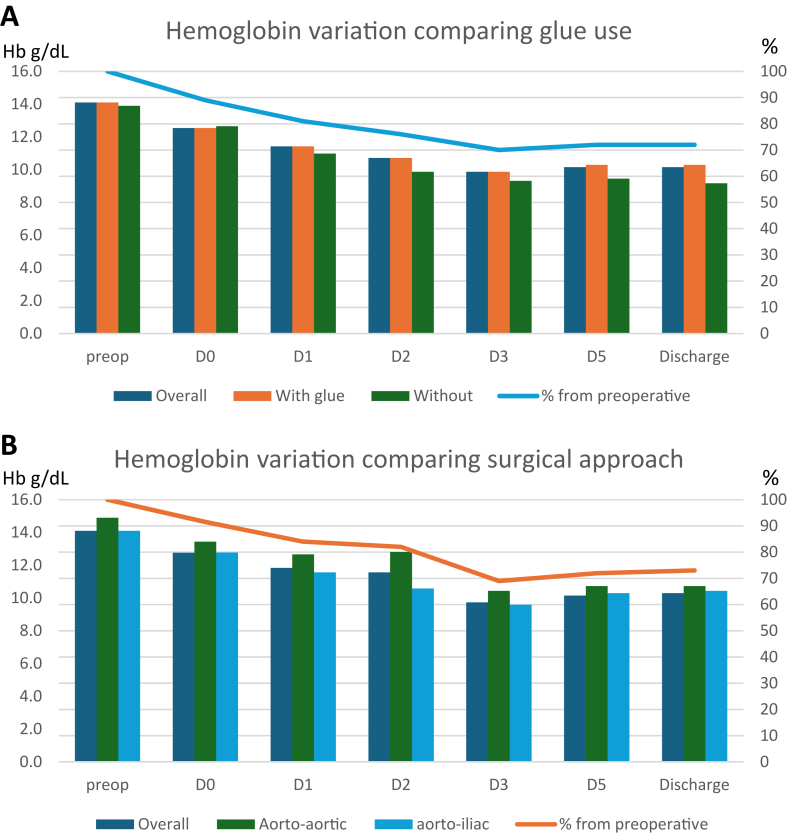
Hemoglobin variation comparing the use of glue (**A**) and the surgical approach (**B**). Hemoglobin (*Hb*) variation. *Dx*, day no.

Hemoglobin details and transfusion management are summarized in Table III. Fig 2, *A*, shows the hemoglobin evolution during the first days. The hemoglobin loss ratio increased from day 0 (the day of surgery) to day 1 (−11% to −19%). These values are primarily due to hemodilution (perioperative hyperhydration of >3 L) rather than major bleeding. The hemoglobin loss ratio was significantly lower at hospital discharge for patients who received surgical glue (−27% vs −34%; *P* = .05), but no significant differences were observed on the preceding days. Postoperative blood transfusion rates did not differ between the groups. Except in cases of active bleeding, the transfusion threshold is 7 g/dL, or 8 g/dL in patients with coronary artery disease.

**Table III:** Perioperative hemoglobin analysis and management of all included patients and comparison if use of glue

Variable	Overall (n = 159)	With glue (n = 130)	Without glue (n = 29)	*P* value
Preoperative Hb, g/dL	14.1 [2.2]	14.1 [2.2]	13.9 [2.4]	.71
Hb ratio preoperative - D0	−11% [10%]	−11% [1%]	−9% [1%]	.27
Hb ratio preoperative - D1	−19% [20%]	−19% [19%]	−21% [18%]	.25
Hb ratio preoperative - D2	−24% [23%]	−24% [23%]	−29% [23%]	.12
Hb ratio preoperative - D3	−30% [22%]	−30% [22%]	−33% [31%]	.14
Hb ratio preoperative - D5	−28% [20%]	−27% [20%]	−32% [24%]	.13
Hb ratio preoperative - discharge	−28% [18%]	−27% [19%]	−34% [17%]	**.05**
Discharge Hb, g/dL	10 [1.7]	10 [1.6]	10 [1.8]	.67
Perioperative blood transfusion	27 (17)	20 (15)	7 (24)	.28
Perioperative plasma transfusion	37 (23)	29 (22)	8 (28)	.54
Perioperative blood restitution	488 [585]	505 [565]	460 [705]	.22
Postoperative transfusion	27 (17)	23 (18)	4 (14)	.79

*Hb,* Hemoglobin.

Values are median [interquartile range] or number (%).

There was no significant difference between the groups concerning early follow-up (<30 days). Follow-up details are shown in Table IV. Six patients required reintervention: three for hemostasis, two for acute ischemia owing to vascular thrombosis related to a fibrinotic embolus, and one for both ischemia and hemorrhage. Thrombectomy was always performed via surgical femoral cutdown. Reinterventions occurred on the day of surgery each time. No bypass was required.

**Table IV:** Early postoperative complications of all included patients and comparison if use of glue

Variable	Overall (n = 159)	With glue (n = 130)	Without glue (n = 29)	*P* value
AKI	61 (38)	51 (39)	10 (34)	.63
AKI requiring ERE	11 (7)	7 (5.4)	4 (14)	.12
Reintervention	6 (4)	4 (3.1)	2 (6.9)	.31
Infection	0 (0)	0 (0)	0 (0)	
Bowel ischemia	0 (0)	0 (0)	0 (0)	
Thrombectomy	3 (2)	3 (2.3)	0 (0)	1
Bypass	0 (0)	0 (0)	0 (0)	
Hemostasis	4 (3)	2 (1.5)	2 (6.9)	.15
Time to reintervention, days	0 [0]	0 [0]	0 [0]	
Early death	5 (3)	3 (2.3)	2 (6.9)	.23

*AKI,* Acute kidney injury; *ERE,* extrarenal epuration.

Values are number (%) or median [interquartile range].

Five patients (3%) died during the 30 days after surgery: one from multivisceral failure on day 1, three from cardiorespiratory arrest on days 7, 12, and 14 (two at home and one before discharge from a rehabilitation center), and one owing to complications related to colorectal cancer on day 22. No patient required reintervention for early infection or bowel ischemia.

### Comparison in glued patients based on type of repair

#### Demographics

Following the flowchart (Fig 1), we compared the impact of the type of vascular repair among patients who received glue. Demographic details are presented in Table V. There was no significant difference between the groups.

**Table V:** Demographic details of all glued patients and comparison between aortic repairs

Variable	Glued (n = 130)	Aortoaortic (n = 82)	Aortoiliac (n = 48)	*P* value
Age, years	70 ± 6.5	70.5 ± 9.9	69.8 ± 5.9	.54
Male	121 (93)	74 (90)	47 (98)	.15
Hypertension	77 (59)	48 (59)	29 (60)	.83
Atrial fibrillation	9 (7)	7 (8; 5)	2 (4.2)	.48
Coronary disease	45 (35)	33 (40)	12 (25)	.08
Renal insuffiency^a^	23 (18)	15 (18)	8 (17)	.81
Diabetes mellitus	28 (22)	16 (20)	12 (25)	.46
PAD	15 (12)	9 (11)	6 (12)	.79
Abdominal surgery	53 (41)	35 (43)	18 (38)	.56
ASA 3 or 4	69 (53)	47 (57)	22 (46)	.21
APT	103 (79)	64 (78)	39 (81)	.66
Oral anticoagulation	15 (12)	12 (15)	3 (6.2)	.15
DOAC	14 (11)	11 (13)	3 (6,2)	.2
VKA	1 (1)	1 (1.2)	0 (0)	1

*APT,* Antiplatelet therapy; *ASA,* American Society of Anesthesiologists; *DOAC,* direct oral anticoagulants; *PAD,* peripherical arterial disease; *VKA,* vitamin K antagonists.

Values are mean ± standard deviation or number (%).

a Glomerular filtration rate of <30 mL/min/1.73 m^2^.

#### Operative details

Operative details are presented in Table VI. As expected, no aortoaortic repair was performed in cases of aortoiliac aneurysms. Retroperitoneal approach was significantly more common in aortoaortic repairs (65% vs 10%; *P* < .001), and a median laparotomy was more frequently used for aortoiliac repairs (90% vs 35%; *P* < .001). The duration of surgery was significantly shorter in aortoaortic repairs: 123 minutes vs 149 minutes (*P* < .001).

**Table VI:** Operative details of all glued patients and comparison between aortic repairs

Variable	All glued (n = 130)	Aortoaortic (n = 82)	Aortoiliac (n = 48)	*P* value
Maximal diameter, mm	56.1 ± 7.3	55.8 ± 5.8	56.5 ± 9.5	.64
Including iliac artery	26 (20)	0 (0)	26 (54)	**<.001**
Laparotomy	72 (55)	29 (35)	43 (90)	**<.001**
Retroperitoneal approach	58 (45)	53 (65)	5 (10)	**<.001**
Duration of surgery, minutes	127 ± 55.8	123 ± 38.7	149 ± 38.6	**<.001**
Duration of clamping, minutes	54.6 ± 20.4	48.9 ± 15.5	64.4 ± 23.9	**<.001**

Values are mean ± standard deviation or number (%).

Boldface entries indicate statistical significance.

#### Early follow-up

Hemoglobin details and transfusion management are summarized in Table VII. Fig 2, *B*, shows the hemoglobin variation during the first days. There was no difference in hemoglobin loss during the hospital stay, nor in transfusion requirements. There was also no significant difference between the groups in terms of reintervention rates, although all reinterventions occurred in the aortoaortic group. Three patients died (3.7%), all in the aortoaortic group. Follow-up details are shown in Table VIII. In our study, systematic duplex control did not reveal any abnormalities at the aortic repair site in any of the patients, with no reductions in outer diameter, lumen diameter, or lumen area.

**Table VII:** Perioperative hemoglobin analysis and management of all glued patients and comparison between aortic repairs

Variable	All glued (n = 130)	Aortoaortic (n = 82)	Aortoiliac (n = 48)	*P* value
Preoperative Hb, g/dL	14.1 ± 2.2	14.9 ± 1.8	14.1 ± 1.9	.53
Hb ratio preoperative - D0	−9.6% ± 12%	−9.8% ± 14%	−9.4% ± 11%	.22
Hb ratio preoperative - D1	−16% ± 21%	−15% ± 22%	−18% ± 17%	.35
Hb ratio preoperative - D2	−18% ± 20%	−14% ± 20%	−25% ± 15%	.24
Hb ratio preoperative - D3	−31% ± 26	−30% ± 28%	−32% ± 23%	.67
Hb ratio preoperative - D5	−28% ± 22%	−28% ± 25%	−27% ± 16%	.76
Hb ratio preoperative - Discharge	−27% ± 23%	−28% ± 27%	−26% ± 14%	.5
Hospital discharge Hb, g/dL	10 ± 1.6	10.6 ± 1.54	10.2 ± 1.25	.12
Perioperative blood transfusion	20 (15)	12 (15)	8 (17)	.76
Perioperative plasma transfusion	29 (22)	17 (21)	12 (25)	.57
Perioperative blood restitution	612 ± 552	578 ± 517	673 ± 612	.38
Postoperative transfusion	23 (18)	15 (18)	8 (17)	.81

*Hb,* Hemoglobin.

Values are mean ± standard deviation or number (%).

**Table VIII:** Early postoperative complications of all glued patients and comparison between aortic repairs

Variable	All glued (n = 130)	Aortoaortic (n = 82)	Aortoiliac (n = 48)	*P* value
AKI	51 (39)	33 (40)	18 (38)	.76
AKI requiring ERE	7 (5)	2 (2.4)	5 (10)	.1
Reintervention	4 (3)	4 (4.9)	0 (0)	.3
Infection	0 (0)	0 (0)	0 (0)	
Bowel ischemia	0 (0)	0 (0)	0 (0)	
Thrombectomy	3 (2)	3 (3.7)	0 (0)	.3
Bypass	0 (0)	0 (0)	0 (0)	
Hemostasis	2 (2)	2 (2.4)	0 (0)	.53
Time to reintervention, days	0 ± 0	0 ± 0	0 ± 0	
Early death	3 (2)	3 (3.7)	0 (0)	.3

*AKI,* Acute kidney injury; *ERE,* extrarenal epuration.

Values are number (%) or mean ± standard deviation.

## Discussion

From 2021 to 2023, 383 patients underwent open aortic surgery at a single referral center. Among these, 159 patients underwent elective aortic aneurysm repair, with 130 patients (82%) receiving glue on the anastomosis.

In our center, the use of glue is not standardized; it is applied at the surgeon's discretion. Among our surgeons, glue is generally either used systematically or not at all, in contrast with Surgicel, which is applied in cases of minor oozing. There are no guidelines or clear indications on when to use glue on the anastomosis: should it be used to achieve hemostasis in cases of coagulopathy, to reinforce a thin and friable aortic wall or suture,^3^ or in cases of low patient temperature?

The only significant difference noted between the use of glue and nonuse is in the hemoglobin ratio at hospital discharge, with no significant impact on transfusion requirements or reintervention rates. Nevertheless, there is a notable trend favoring the use of glue, with lower rates of reintervention for hemostasis (1.5% vs 6.9%; *P* = .15) and lower mortality (2.3% vs 6.9%; *P* = .23). There is also a trend toward less postoperative blood loss, reflected in the hemoglobin ratio.

When comparing types of aortic repair, significant differences were observed in surgical strategy and aneurysm location. Anatomic considerations, assessed preoperatively via CT scan, guide the choice of surgical approach. For aortic surgery, a retroperitoneal approach is typically performed on the left, whereas anastomosis on the right iliac can be challenging, which may explain why most aneurysms, including aortoiliac aneurysms, are managed via laparotomy. No significant differences in follow-up outcomes were observed between the types of repairs. More aortoaortic repairs are performed using a retroperitoneal approach. Patients treated via the retroperitoneal approach are glued more often than those treated via a midline laparotomy. Therefore, there is a bias related to the surgical approach affecting the duration of surgery.

We report a slightly higher rate of reintervention for hemostasis than that reported in the literature by Deery et al^3^ and Milne et al^4^ (3.0% vs 1.5%–1.7%), but a lower overall reintervention rate (4.0% vs 7.1%), particularly for major abdominal surgery (0% vs 2.6%).

This study was conducted in a center where glue is frequently used across various types of vascular surgeries, including aortic procedures (as presented here), carotid surgeries, and peripheral interventions, and has been in use for a long time. Nevertheless, the decision to use glue remains surgeon dependent. The rationale for glue use is not well-defined; there are no clear guidelines available in the literature. Although many studies examine the use of surgical adhesives in cardiac surgery (such as in ascending aorta and aortic root procedures), literature on its use in abdominal aortic surgeries is limited, particularly concerning Ifabond. According to the instructions for use, the glue is indicated for “its adhesive action and its role as hemostatic sealant agent. IFABOND is intended for use in open and laparoscopic surgery: Mesh fixation in digestive surgery for treatment of hernia and in uro-gynecological surgery for sacrocolpopexy. Staple-line reinforcement in bariatric surgery for sleeve and closure of mesenteric defects in bariatric surgery for bypass.” Contraindications are as follows:

The glue should not be applied on fragile and/or damaged tissue that may not withstand thermal, inflammatory or mechanical stress. The use of glue is not suitable for ophthalmic surgery with PETERS SURGICAL's existing applicators. The glue should not be applied on cerebral tissue. Use of the glue is contra-indicated in orthopedic surgery. The glue should not be applied to incisions or openings with significant tension without being combined with other fixation or closure methods. Use of the glue is contra-indicated for any infected wound or where there is a risk of gangrene. The glue should not be used on patients with a known hypersensitivity to cyanoacrylate and/or its related degradation products. The glue should not be used on pregnant women, or on patients with a systemic pre-operative infection, with uncontrolled diabetes, or with conditions known to interfere with the healing process.

Pacini et al^5^ studied the in vitro effects of glue on polypropylene sutures and found no increase in suture breakage with glue application.

LeMaire et al^6^ studied the use of glue in young pigs. Histological analysis revealed extensive fibrosis adjacent to the anastomosis and widespread adventitial changes characterized by a moderate increase in connective tissue, dense macrophage infiltration, micropyogranulomas, and dystrophic calcification. After the pigs' growth, animals treated with BioGlue showed significantly increased stenosis, with reductions in outer diameter (14.4%), lumen diameter (19%), and lumen area (33.9%).^6^ This should be taken into consideration for children, but is unlikely to have a significant impact on the adult population. In this aortic study, no decreases in outer diameter, lumen diameter, or lumen area were observed.

In a study on sheep with induced coagulopathy conducted by Hewitt et al,^7^ the use of glue significantly reduced intraoperative and postoperative blood loss compared with the use of Surgicel. Ma et al^8^ analyzed postoperative CT scans of thoracic aortic surgeries with glue application and found no association with an increased incidence of anastomotic pseudoaneurysms.

In a prospective, multicenter, randomized, controlled clinical trial of 151 patients, Coselli et al^9^ studied the impact of glue vs no glue on anastomosis during cardiac, aortic, and peripheral surgeries. Anastomotic bleeding was significantly reduced in the BioGlue group (18.8% vs 42.9%; *P* < .001), as was the use of pledgets (26.2% vs 35.9%; *P* = .047).^9^

Some studies report side effects associated with the use of biological glue. Potential neurotoxicity has been demonstrated when BioGlue (purified bovine serum albumin and glutaraldehyde) is applied directly to nerves (as evidenced by testing diaphragmatic excursion while stimulating the phrenic nerve).^10^ Application of Bioglue to lung and liver tissue appears to induce high-grade inflammation and lead to cytotoxic reactions. These reactions have not been reported in aortic tissue.^11^

When comparing different types of glue, stiffness varies. Sealants that are stiffer than the replacement material may restrict normal physiological dilation and lead to anastomotic strictures.^12^ There are plenty of hemostatic agents,^13^ including foam and Surgicel, that have been associated with reduced blood loss.^14^ Fibrillar Surgicel is indeed a widely used material owing to both its chemical and physical properties as a packing agent.

Meticulous perioperative hemostasis is a key factor in preventing major bleeding and the need for reintervention. Nevertheless, perioperative management by the anesthetist and in the intensive care unit is also important. The significance of vasopressor drugs has been demonstrated in maintaining hemodynamic stability, especially during aortic unclamping; however, this must be complemented by appropriate fluid and transfusion management, including fluid restriction and blood conservation techniques such as cell salvage. Tranexamic acid is used in all patients. Patients with coagulopathy should be monitored with thromboelastography and treated with blood products and factor VIIa, if necessary.^15^ The use of fresh frozen plasma and platelet transfusions depends on preoperative coagulopathy and on the occurrence of massive transfusion. They were not necessary in the patients included in this report. Patients requiring reintervention for hemostasis had no major coagulopathy (mean activated partial thromboplastin time = 57 seconds, anti-Xa = 0.34 IU/mL) nor acidosis (worst mean arterial values: lactate = 2.5 mmol/L, pH = 7.31, bicarbonate = 22.13 mmol/L). The use of effective pain management during and after surgery has also been shown to impact survival and postoperative outcomes.^16^

To achieve effective surgical and anesthetic management and cooperation, it is recommended that open surgery be performed in high-volume centers, with a proposed minimum threshold of 6 to 10 open aortic surgeries per year.^17^^,^^18^ Although this threshold may seem low, the number of high-volume centers adhering to these guidelines is decreasing, likely owing to the increasing prevalence of EVAR.^19^ This finding underscores the importance and impact of regionalization in open aortic surgery. It also allows high-volume centers to treat evolving abdominal aneurysms after EVAR with acceptable mortality rates (2.7% for elective treatment), despite the older age and increased comorbidity burden among these patients, along with the more complex nature of these surgeries.^20^

Although there are only a few reinterventions reported in our series, we included only patients with exclusive intraabdominal repair and early follow-up. We excluded aortofemoral repairs because the demographic details of these patients are more similar to those with peripheral artery disease (PAD) than to patients undergoing aortoaortic or aortoiliac repairs. Indeed, patients treated for PAD with aortofemoral bypass tend to present more postoperative comorbidities, including infections associated with groin access (requiring reintervention or not), as well as more postoperative reinterventions for thrombectomy or femoropopliteal bypass owing to associated PAD. Moreover, the aortic wall in these patients is more calcified, making it harder to suture and more friable, which may lead to a higher risk of emboli and a more porous anastomosis.

Three patients required a thrombectomy after surgery. All had undergone aortoaortic repair and received glue at the anastomosis. It is unlikely that the glue crossed the suture and caused a migrating clot: in the case of an impervious suture, this seems illogical, and if there is blood oozing, the glue would be pushed away by the blood rather than crossing the suture.

Some limitations can be cited, such as the single-center, retrospective, and descriptive nature of the study. This review would indeed benefit from a larger number of patients, which could be achieved by including patients from multiple centers with dedicated intensive care units, as is the case in our center. This strategy could render some statistical trends significant. In case of multicentric inclusion, there may be a bias in perioperative care if the anesthetists or intensivists are not specialized, because perioperative management has been shown to play a key role in decreasing perioperative morbidity. The use of Surgicel is not clearly reported, and the analyses cannot be stratified based on this variable, which introduces a bias since it is a hemostatic agent.

Other limitations include the short follow-up period and the selective criteria for patient inclusion. We analyzed only early follow-up outcomes, because the main objective was to assess the impact of surgical glue use on hemorrhage and reintervention, which typically occurs within the first days after surgery. As described by Kieffer et al,^21^ we do encounter late reinterventions for hernias or other complications linked to laparotomy. For this reason, a retroperitoneal approach is widely encouraged when possible in our center. Further studies comparing postoperative parietal reinterventions between retroperitoneal approach and laparotomy should be conducted.

We excluded aortic repairs involving aortofemoral bypass because the patient demographics are more similar to those of PAD patients, who have inherent comorbidities and require femoral surgical cutdown, which is associated with intrinsic infectious risks and complications related to wound healing. We also excluded emergent and infectious procedures owing to concerns regarding coagulopathy, systemic inflammation, and the sometimes severe conditions at hospital arrival, which directly impact hemostasis. Additionally, we excluded visceral bypasses and nonpararenal aneurysms because the duration of surgery and aortic cross-clamping is longer in higher aortic zones, leading to increased ischemic time in abdominal organs (liver, bowel, and kidneys) and the limbs, resulting in greater systemic stress.

Further studies, such as randomized multicenter trials involving a larger number of patients, should be conducted to explore the trends highlighted in this study. This work could help to precisely determine the benefits of surgical adhesive use and clarify its role in the hemostatic arsenal for abdominal aortic surgery.

## Conclusions

Reinterventions after open aortic surgeries are marked by high mortality and morbidity rates. One of the most frequent causes is bleeding. Hemostasis can be facilitated by optimal medical management as well as by surgical adjuncts such as adhesive. The current literature on the use of glue in abdominal aortic surgeries is scarce.

This retrospective study, involving 159 patients undergoing abdominal aortic surgery, found that 82% received glue on their aortic anastomosis, which was associated with a slight decrease in reintervention rates for hemostasis and postoperative blood loss, but had no effect on blood transfusion requirements. However, the data are equivocal, and the clinical benefit of glue in abdominal aortic surgery requires further investigation in larger patient populations to be defined.

## Author Contributions

Conception and design: CB, LC

Analysis and interpretation: CB, KH, PA

Data collection: CB, AZ

Writing the article: CB, AZ, LC

Critical revision of the article: CB, KH, PA, LC

Final approval of the article: CB, AZ, KH, PA, LC

Statistical analysis: CB

Obtained funding: Not applicable

Overall responsibility: CB

## Funding

None.

## Disclosures

None.
